# Supplementary material for: Women’s Health: Contemporary Management of MS in Pregnancy and Post-Partum
Source: Biomedicines. 2019 Apr 19;7(2):32. doi: 10.3390/biomedicines7020032 (PMC6630249; doi:10.3390/biomedicines7020032)
Supplement: Supplementary file 1 [file biomedicines-07-00032-s001.docx]

**Supplementary Material: Table S1.** DMT use in Pregnancy: Summary of Risk to Fetus From DMT Label, With Updated Registry Data and Other Sources.

| **DMT Name** | **Summary of Risk to Fetus** |
| --- | --- |
| Interferon Beta | *Animals*: Dose dependent abortifacient activity[1].  *Humans*: There are no adequate and well-controlled studies in pregnant women. Early data concerning for preterm birth, shorter mean birth length, and lower mean birth weight[2].  German Multiple Sclerosis and Pregnancy Registry (96 pregnancies) and Betaseron pregnancy registry (251 live births): data shows early exposure to interferon beta is not associated with increased risk of teratogenic or abortive effects or adverse effects on pregnancy outcomes[2-4]. |
| Glatiramer Acetate | *Animals:* No evidence of negative effects[5].  *Humans:* Early data showed no evidence of harm in early pregnancies exposed to glatiramer acetate[5].  German MS and Pregnancy Registry (151 exposed pregnancies) data show that early exposure to glatiramer acetate was not associated with an increased risk of adverse pregnancy outcomes when compared to the general population[2,6].  Teva pharmacovigilance database (over 5042 pregnancies) confirmed that early exposure to branded glatiramer acetate was not associated with a higher risk of congenital malformations when compared to reference US and European population[2,7]. |
| Fingolimod | *Animals:* Fingolimod demonstrated an increase in malformations and embryolethality, when given to pregnant animals. The sphingosine 1-phosphate receptor is known to be involved in vascular formation during embryogenesis[8].  *Humans:* There are no adequate data on the developmental risk associated with the use of fingolimod in pregnant women[8].  Fingolimod clinical development program (89 pregnancies) data found a possible increase in spontaneous abortion and abnormal fetal development[2].  Data from the Novartis Safety Database (NSD), Gilenya Pregnancy Exposure Registry (Registry), and Pregnancy outcomes intensive Monitoring (PRIM) compared outcomes of fingolimod-exposed MS pregnancies to disease-matched unexposed MS pregnancies and to pregnancies in the general population and found no difference in the prevalence of major congenital malformations or  miscarriages. The prevalence of major cardiac malformations was not statistically significantly higher than the general population[9]. |
| Dimethyl Fumarate | *Animals:* Reproductive Toxicity[10].  *Humans:* There are no adequate data on the developmental risk associated with the use of dimethyl fumarate in pregnant women[10].  No increased risk in fetal harm or adverse pregnancy outcomes in over 200 pregnancies from data reported from the post-marketing setting[2].  A report on a conference-abstract with preliminary data from the Biogen Multiple Sclerosis Pregnancy Exposure Registry described 48 pregnancies exposed to dimethyl fumarate reported that in 48 exposed pregnancies, outcomes for 16 fetuses were known. One spontaneous abortion, two premature births, and no birth defects or neonatal deaths were reported[2]. |
| Teriflunomide | *Animals:* During organogenesis, teriflunomide caused teratogenicity and embryolethality[11].  *Humans:* Inadequate human data available to inform the presence or absence of drug-associated risk with the use of teriflunomide during pregnancy[11].  The Global Pharmacovigiliance database retrospectively analyzed participants from in the teriflunomide clinical trials including 83 female pregnancies and 22 pregnancies in partners of male patients treated with teriflunomide. No congenital abnormalities were found. The spontaneous abortion rate was similar to the reported rate of the general population[12,13]. |
| Natalizumab | *Animals:* Fetal immunologic and hematologic effects were seen in monkeys, and reduced pup survival in guinea pigs[14].  *Humans:* Inadequate human data in pregnancy exists[14].  German Pregnancy Registry: 101 exposed German MS women with MS were compared to 78 Canadian disease matched (DM) unexposed controls and to 97 Canadian health controls (HC). No significant differences in rates of major congenital malformations, premature births, or low birth weights were seen. The exposed and DM groups had higher rates of spontaneous abortion compared to the HC group[2,15].  Case Reports: Transient hematologic abnormalities were reported in newborns born to women with exposure to natalizumab during pregnancy. Discontinuation of natalizumab at 30 weeks may reduce this risk, but requires further evaluation[2]**.**  Global Tysabri Pregnancy Exposure Registry Database: In 363 exposed pregnancies for patients treated for MS or Cronhs Disease (CD), women with early exposure to natalizumab had an increased rate of major malformations as compared to the reference US general population. 26 major and minor malformations were possibly temporally related to natalizumab, but no specific pattern of defect was found to suggest drug effect. The spontaneous abortion rate was similar to the rate reported in the general population[2,16]. |
| Alemtuzumab | *Animals:* Alemtuzumab was embryolethal in pregnant huCD52 transgenic mice[17].  *Humans:* Human IgG is known to cross the placenta and thus, alemtuzumab may cross, potentially causing fetal harm. Alemtuzumab may cause maternal autoimmune thyroid disease and if untreated in pregnancy, hypothyroidism may cause miscarriage, fetal mental retardation, and fetal dwarfism. Maternal auto-antibodies may develop after administration of alemtuzumab and placental transfer of anti-thyroid antibodies resulting in neonatal Graves’ disease has been reported[17].    Pooled data from several Phase 2 studies and a post-trial extension phase: In 183 exposed pregnancies, 66% were live births without birth defects and the spontaneous abortion rate was 22%, similar to the general population or untreated MS patients[2,18].  German MS and Pregnancy Registry: In 7 exposed (treated a median of 42 days prior to LMP) and 8 possibly exposed (treated a median of 168 days prior to LMP) pregnancies, one child was born with a single kidney and hydronephrosis, and one child born with hypospadias. There were no preterm births[2]. |
| Ocrelizumab | *Animals:* Increased perinatal mortality, depletion of B-cell populations, renal, bone marrow, and testicular toxicity were observed in the offspring of pregnant monkeys[19].  *Humans:* Ocrelizumab is a humanized monoclonal IgG1 antibody, a subtype which is known to cross the placental barrier. There are no adequate data on the developmental risk associated with use of Ocrelizumab in pregnant women. However, transient peripheral B-cell depletion and lymphocytopenia have been reported in infants born to mothers exposed to other anti-CD20 antibodies during pregnancy. B-cell levels in infants following maternal exposure to ocrelizumab have not been studied in clinical trials. The potential duration of B-cell depletion in such infants, and the impact of B-cell depletion on vaccine safety and effectiveness, is unknown[19].  No registry data is currently available. |
| Mitoxantrone | *Animals:* Fetal growth retardation and increased incidence of premature delivery was observed in animals[20].  *Humans:* Mitoxantrone is considered a potential human teratogen because of its mechanism of action and the developmental effects demonstrated by related agents. There are no adequate and well-controlled studies in pregnant women[20].  Three reported cases: 1 case of fetal growth restriction without malformations in pregnancy with mitoxantrone exposure until 29 weeks gestation for maternal MS[21], 1 case of no malformation in pregnancy with mitoxantrone exposure during week 22 for maternal AML relapse[22], and 1 case of Pierre Robin syndrome in a child born to a mother treated with mitoxantrone 8-9 weeks prior to presumed conception for maternal MS[23]. |
| Rituximab (Not FDA approved for treatment of MS) | *Animals:* In cynomolgus monkeys, rituximab caused lymphoid B-cell depletion in the offspring when administered during organogenesis[24].  *Humans:* Rituximab can cause adverse developmental outcomes including B-cell lymphocytopenia in infants exposed to rituximab in-utero[24].  Rituximab Global Safety Database 2011: Pregnancy in rituxmab exposed patients treated for MS, Rheumatoid Arthritis, Systemic Lupus Erythematosus, Lymphoma, Immune Thrombocytopenic Purpura/Thrombotic Thrombocytopenic Purpura, and Castleman disease were reported[25]. No increase in congenital malformations was found. Higher rates of preterm delivery and early pregnancy loss compared to the general population, but the rates in those with chronic disease, auto-immune diseases, and malignancy is unknown, and these patients are often treated with other teratogenic medications[25].  Das et al found 102 women treated with rituximab within 6 months prior to conception or during pregnancy for various medical conditions. The spontaneous abortion rate was 12%, premature delivery rate was 41%, malformation rate was 4.5%. 39% of newborns developed transient low B-cell counts which returned to normal in 6 months. Within this review, a case series of 11 pregnancies in 10 women treated for MS (7) or NMOSD (3) was included, and outcomes for 9 pregnancies were known, with no adverse outcomes[26]. |
| Cyclophosphamide (Not FDA approved for treatment of MS) | *Animals:* Exposure to cyclophosphamide during pregnancy may cause fetal malformations, miscarriage, fetal growth retardation.  *Humans:*  Cyclophosphamide can cause fetal harm when administered to a pregnant woman. Common fetal malformations include hydrocephalus, microretrognathia, ectrodactyly, cleft palate, and exencephaly[27]. |

References:

1. AVONEX (interferon beta-1a) intramuscular injection. Highlights of prescribing information. Availabe online: <https://www.avonex.com/content/dam/commercial/multiple-sclerosis/avonex/pat/en_us/pdf/Avonex_US_Prescribing_Information.pdf> (accessed on 29 January 2019).

2. Vaughn, C.; Bushra, A.; Kolb, C.; Weinstock-Guttman, B. An Update on the Use of Disease-Modifying Therapy in Pregnant Patients with Multiple Sclerosis. *CNS Drugs* **2018**, *32*, 161-178, doi:10.1007/s40263-018-0496-6.

3. Thiel, S.; Langer-Gould, A.; Rockhoff, M.; Haghikia, A.; Queisser-Wahrendorf, A.; Gold, R.; Hellwig, K. Interferon-beta exposure during first trimester is safe in women with multiple sclerosis-A prospective cohort study from the German Multiple Sclerosis and Pregnancy Registry. *Mult Scler* **2016**, *22*, 801-809, doi:10.1177/1352458516634872.

4. Coyle, P.K.; Sinclair, S.M.; Scheuerle, A.E.; Thorp, J.M., Jr.; Albano, J.D.; Rametta, M.J. Final results from the Betaseron (interferon beta-1b) Pregnancy Registry: a prospective observational study of birth defects and pregnancy-related adverse events. *BMJ Open* **2014**, *4*, e004536, doi:10.1136/bmjopen-2013-004536.

5. Glatiramer acetate  (glatiramer acetate injection) for subcutaneous use. Highlights of prescribing information. . Availabe online: <https://www.copaxone.com/Resources/pdfs/PrescribingInformation.pdf> (accessed on 29 January 2019).

6. Herbstritt, S.; Langer-Gould, A.; Rockhoff, M.; Haghikia, A.; Queisser-Wahrendorf, A.; Gold, R.; Hellwig, K. Glatiramer acetate during early pregnancy: A prospective cohort study. *Mult Scler* **2016**, *22*, 810-816, doi:10.1177/1352458515623366.

7. Sandberg-Wollheim, M.; Neudorfer, O.; Grinspan, A.; Weinstock-Guttman, B.; Haas, J.; Izquierdo, G.; Riley, C.; Ross, A.P.; Baruch, P.; Drillman, T., et al. Pregnancy Outcomes from the Branded Glatiramer Acetate Pregnancy Database. *Int J MS Care* **2018**, *20*, 9-14, doi:10.7224/1537-2073.2016-079.

8. Gilenya (fingolimod) capsules, for oral use. Highlights of prescribing information. Availabe online: <https://www.pharma.us.novartis.com/sites/www.pharma.us.novartis.com/files/gilenya.pdf> (accessed on 29 January 2019).

9. Geissbuhler, Y.; Vile, J.; Koren, G.; Guennec, M.; Butzkueven, H.; Tilson, H.; MacDonald, T.M.; Hellwig, K. Evaluation of pregnancy outcomes in patients with multiple sclerosis after fingolimod exposure. *Ther Adv Neurol Disord* **2018**, *11*, 1756286418804760, doi:10.1177/1756286418804760.

10. Tecfidera (dimethyl fumarate) delayed-release capsules, for oral use. Highlights of prescribing information. . Availabe online: <https://www.tecfidera.com/content/dam/commercial/multiple-sclerosis/tecfidera/pat/en_us/pdf/full-prescribing-info.pdf> (accessed on 28 January 2019).

11. Aubagio (teriflunomide) tablets, for oral use. Highlights of prescribing information. Availabe online: <http://products.sanofi.us/aubagio/aubagio.pdf> (accessed on 28 January 2019).

12. Kieseier, B.C.; Benamor, M. Pregnancy outcomes following maternal and paternal exposure to teriflunomide during treatment for relapsing-remitting multiple sclerosis. *Neurol Ther* **2014**, *3*, 133-138, doi:10.1007/s40120-014-0020-y.

13. Voskuhl, R.; Momtazee, C. Pregnancy: Effect on Multiple Sclerosis, Treatment Considerations, and Breastfeeding. *Neurotherapeutics* **2017**, *14*, 974-984, doi:10.1007/s13311-017-0562-7.

14. Tysabri (natalizumab) injection, for intravenous use. Highlights of prescribing information. Availabe online: <https://www.tysabri.com/content/dam/commercial/multiple-sclerosis/tysabri/pat/en_us/pdfs/tysabri_prescribing_information.pdf>. (accessed on 29 January 2019).

15. Ebrahimi, N.; Herbstritt, S.; Gold, R.; Amezcua, L.; Koren, G.; Hellwig, K. Pregnancy and fetal outcomes following natalizumab exposure in pregnancy. A prospective, controlled observational study. *Mult Scler* **2015**, *21*, 198-205, doi:10.1177/1352458514546790.

16. Friend, S.; Richman, S.; Bloomgren, G.; Cristiano, L.M.; Wenten, M. Evaluation of pregnancy outcomes from the Tysabri(R) (natalizumab) pregnancy exposure registry: a global, observational, follow-up study. *BMC Neurol* **2016**, *16*, 150, doi:10.1186/s12883-016-0674-4.

17. Lemtrada (alemtuzumab) injection, for intravenous use. Highlights of prescribing information. Availabe online: <http://products.sanofi.us/lemtrada/lemtrada.pdf?s_mcid=ps-LP-google-BRsitelink-pi> (accessed on 28 January 2019).

18. Alroughani, R.; Altintas, A.; Al Jumah, M.; Sahraian, M.; Alsharoqi, I.; AlTahan, A.; Daif, A.; Dahdaleh, M.; Deleu, D.; Fernandez, O., et al. Pregnancy and the Use of Disease-Modifying Therapies in Patients with Multiple Sclerosis: Benefits versus Risks. *Mult Scler Int* **2016**, *2016*, 1034912, doi:10.1155/2016/1034912.

19. Ocrevus (ocrelizumab) injection, for intravenous use. Highlights of prescribing information. Availabe online: <https://www.gene.com/download/pdf/ocrevus_prescribing.pdf> (accessed on 29 January 2019).

20. Novantrone, mitoxantrone for injection concentrate. Availabe online: <https://www.accessdata.fda.gov/drugsatfda_docs/label/2009/019297s030s031lbl.pdf> (accessed on 29 January 2019).

21. De Santis, M.; Straface, G.; Cavaliere, A.F.; Rosati, P.; Batocchi, A.P.; Caruso, A. The first case of mitoxantrone exposure in early pregnancy. *Neurotoxicology* **2007**, *28*, 696-697, doi:10.1016/j.neuro.2006.10.002.

22. Baumgartner, A.K.; Oberhoffer, R.; Jacobs, V.R.; Ostermayer, E.; Menzel, H.; Voigt, M.; Schneider, K.T.; Pildner von Steinburg, S. Reversible foetal cerebral ventriculomegaly and cardiomyopathy under chemotherapy for maternal AML. *Onkologie* **2009**, *32*, 40-43, doi:10.1159/000184745.

23. Hellwig, K.; Schimrigk, S.; Chan, A.; Epplen, J.; Gold, R. A newborn with Pierre Robin sequence after preconceptional mitoxantrone exposure of a female with multiple sclerosis. *J Neurol Sci* **2011**, *307*, 164-165, doi:10.1016/j.jns.2011.05.003.

24. Rituxan (rituximab) injection, for intravenous use. Highlights of prescribing information. Availabe online: <https://www.gene.com/download/pdf/rituxan_prescribing.pdf> (accessed on 29 January 2019).

25. Chakravarty, E.F.; Murray, E.R.; Kelman, A.; Farmer, P. Pregnancy outcomes after maternal exposure to rituximab. *Blood* **2011**, *117*, 1499-1506, doi:10.1182/blood-2010-07-295444.

26. Das, G.; Damotte, V.; Gelfand, J.M.; Bevan, C.; Cree, B.A.C.; Do, L.; Green, A.J.; Hauser, S.L.; Bove, R. Rituximab before and during pregnancy: A systematic review, and a case series in MS and NMOSD. *Neurology(R) neuroimmunology & neuroinflammation* **2018**, *5*, e453, doi:10.1212/NXI.0000000000000453.

27. Amato, M.P.; Portaccio, E. Fertility, pregnancy and childbirth in patients with multiple sclerosis: impact of disease-modifying drugs. *CNS Drugs* **2015**, *29*, 207-220, doi:10.1007/s40263-015-0238-y.
